# Mandibular Chondrosarcoma Mimicking an Odontogenic Infection: A Case Report

**DOI:** 10.7759/cureus.116429

**Published:** 2026-09-17

**Authors:** Morgane Basic, William Calvin, Jordan Bouchet

**Affiliations:** 1 Oral and Maxillofacial Surgery, Centre Hospitalier Intercommunal de Villeneuve-Saint-Georges, Villeneuve-Saint-Georges, FRA; 2 Oral Surgery, Hôpital Saint-Antoine, Paris, FRA

**Keywords:** chondrosarcoma, fibula free flap, jaw neoplasm, mandible, odontogenic infection, oral surgery

## Abstract

Chondrosarcoma is a malignant mesenchymal tumor exhibiting cartilaginous differentiation. It is a relatively common primary malignant bone tumor, but its occurrence in the jaws is rare, particularly in the mandible. The clinical presentation may be nonspecific and may mimic odontogenic or inflammatory conditions, potentially resulting in delayed diagnosis. We report the case of a 37-year-old woman with mandibular chondrosarcoma initially presenting with persistent pain in the region of the right mandibular first molar. The lesion was initially managed as a dental condition, and endodontic treatment was performed. Despite treatment, the patient developed persistent symptoms, recurrent right buccal cellulitis, progressive mandibular enlargement, and eventually visible facial asymmetry. Imaging demonstrated an approximately 40 mm intramandibular lesion with irregular borders and cortical expansion. Because of repeated loss to follow-up and limited compliance, no preoperative biopsy was performed before the initial surgical procedure. Intraoperative examination revealed a solid tissue-like lesion rather than a conventional odontogenic cyst. Histopathological examination demonstrated an infiltrative neoplasm with cartilaginous differentiation suspicious for chondrosarcoma. Although the definitive resection specimen raised a differential diagnosis of low-grade parosteal osteosarcoma, expert sarcoma review ultimately retained the diagnosis of mandibular chondrosarcoma. Staging investigations revealed no regional nodal or distant metastatic disease, and the tumor was classified as T4N0M0. The patient subsequently underwent interruptive mandibulectomy with tracheostomy and reconstruction using a vascularized free fibula flap. This case highlights the diagnostic challenges associated with rare mandibular malignancies presenting with apparently odontogenic symptoms and recurrent infection.

## Introduction

Chondrosarcoma is a malignant mesenchymal tumor characterized by cartilaginous differentiation and represents one of the most common primary malignant tumors of bone. Most conventional chondrosarcomas arise in the axial skeleton or long bones, whereas involvement of the craniofacial skeleton is uncommon [1,2]. In a review of 224 cases, de Souza et al. reported a slight male predominance, with most patients presenting during the second to fifth decades of life. Swelling was the most common presenting symptom. The maxilla was more frequently affected than the mandible, and surgical resection was the principal therapeutic approach [1]. Earlier clinicopathological series similarly emphasized the rarity and locally aggressive nature of chondrosarcoma affecting the jaw and facial bones [3,4]. The predominance of maxillary involvement makes mandibular chondrosarcoma a particularly uncommon presentation, potentially contributing to diagnostic challenges when lesions mimic odontogenic disease.

The clinical presentation of mandibular chondrosarcoma may be nonspecific. Pain, swelling, facial asymmetry, dental displacement, tooth mobility, malocclusion, or neurological symptoms may occur depending on the location and extent of the tumor [1,2]. Consequently, lesions arising in the posterior mandible may initially be mistaken for odontogenic, periodontal, or inflammatory disease.

Radiological diagnosis may also be challenging. CT and MRI are important for evaluating tumor extent, cortical involvement, mineralized matrix, and soft-tissue extension. However, the radiological appearance of chondrosarcoma is variable and must be interpreted in conjunction with clinical and pathological findings [2,5].

Histopathological diagnosis may be particularly difficult in small or decalcified specimens and requires evaluation of cytological atypia, cartilaginous matrix, growth pattern, and the relationship between the lesion and surrounding bone. Differential diagnoses include benign cartilaginous lesions and osteosarcoma, particularly chondroblastic or surface osteosarcoma [2,6].

We report a case of mandibular chondrosarcoma in a 37-year-old woman initially presenting with persistent dental pain and subsequently complicated by recurrent buccal cellulitis. The case was characterized by delayed progression, misleading odontogenic features, histopathological discordance, and the need for definitive mandibular resection and free fibula reconstruction.

## Case presentation

A 37-year-old woman was referred for the management of a progressively enlarging lesion of the right mandible. Her medical history was notable for asthma. She reported smoking approximately one pack of cigarettes per day, occasional alcohol consumption, and regular evening cannabis use. No other significant medical history was reported.

The patient’s initial symptoms consisted of pain localized to the right posterior mandibular dental region, particularly around tooth 46. The lesion was initially managed as a dental condition, and endodontic treatment of tooth 46 was performed by her general dental practitioner. Despite treatment, the symptoms persisted. Over several months, the patient experienced recurrent episodes of acute right mandibular pain associated with infectious exacerbations and recurrent episodes of right buccal cellulitis. The pain was episodic and resolved between acute episodes, with no persistent pain reported outside these infectious episodes. She presented repeatedly to emergency departments, where some episodes were associated with collections requiring drainage and antibiotic treatment with amoxicillin. Symptoms improved with antibiotic therapy and WHO step II analgesics. This intermittent clinical course and the relief of symptoms following treatment may have contributed to the patient’s limited adherence to follow-up and delayed surgical management. Despite the transient improvement of acute symptoms, the mandibular lesion continued to enlarge over time. The patient eventually developed visible extraoral facial asymmetry due to enlargement of the right mandible, a feature that had not been present initially. Clinical examination revealed no cervical lymphadenopathy. No dental mobility was observed, including at tooth 46 despite the previous endodontic treatment. No sensory or motor neurological deficit was reported.

Radiological findings

The initial radiological assessment demonstrated a right intramandibular radiolucent lesion with homogeneous content, irregular borders, and marked cortical expansion. The lesion measured approximately 40 mm in its greatest dimension. The available panoramic radiograph (Figure 1) and cross-sectional imaging (Figure 2) demonstrated involvement of the right posterior mandible, including the horizontal branch and mandibular angle. The lesion appeared expansile and destructive, with marked alteration of the surrounding cortical bone. Because of repeated loss to follow-up and limited compliance, no preoperative biopsy was performed before the initial surgical procedure.

**Figure 1:**
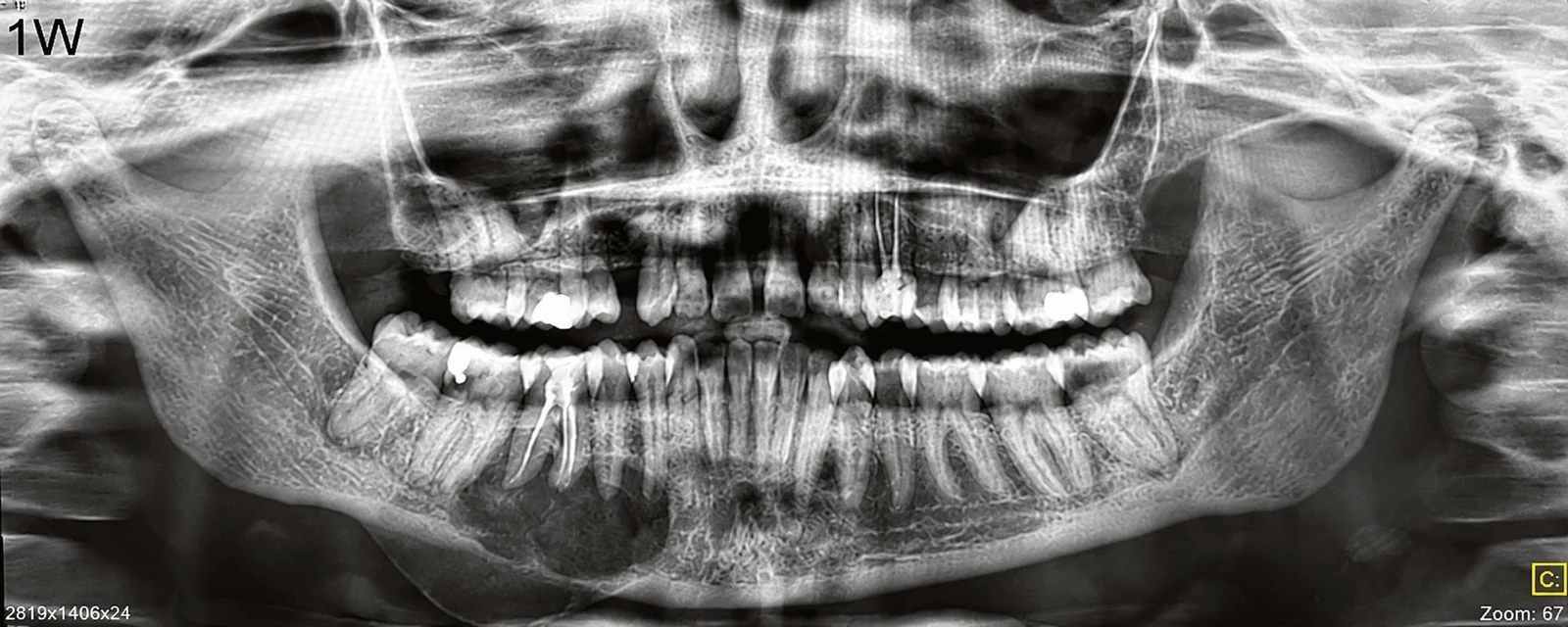
Initial panoramic radiograph showing a radiolucent lesion of the right mandible. The lesion involves the posterior region of the right mandible and is associated with irregular borders and cortical expansion.

**Figure 2:**
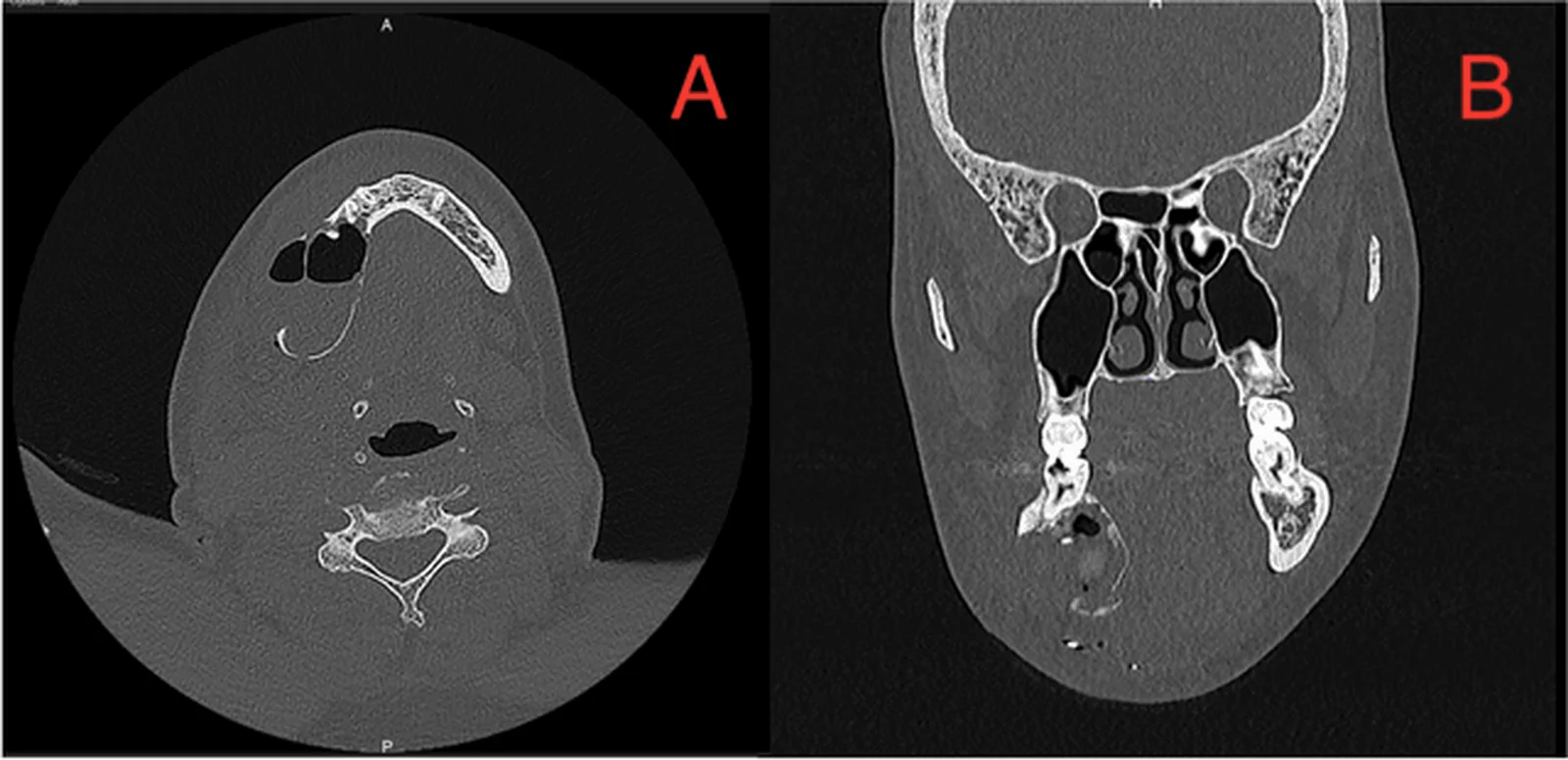
Three-dimensional imaging of the right mandibular lesion. (A) Axial CT section showing the lesion involving the right mandible. (B) Coronal CT section demonstrating the extent of the mandibular lesion and the associated cortical involvement. Maximum lesion dimension: 40 mm.

Initial surgical management and pathological findings

Surgical exploration and excision of the lesion were performed. Intraoperatively, the lesion did not demonstrate the characteristics expected for a conventional odontogenic cyst. Instead, a solid tissue-like proliferation was identified. The intraoperative appearance therefore made a simple dentigerous or inflammatory cyst unlikely (Figures 3, 4). The lesion was submitted for histopathological examination. The intramandibular tumor specimen consisted of a fragment measuring 3.5 × 2.5 × 0.8 cm. A second soft-tissue specimen from the basilar region measured 2.5 × 1 × 1.2 cm. A third specimen of right mandibular bone measured 3 × 0.6 × 0.2 cm. The basilar soft-tissue specimen consisted of a poorly cellular fibrous wall without atypia.

**Figure 3:**
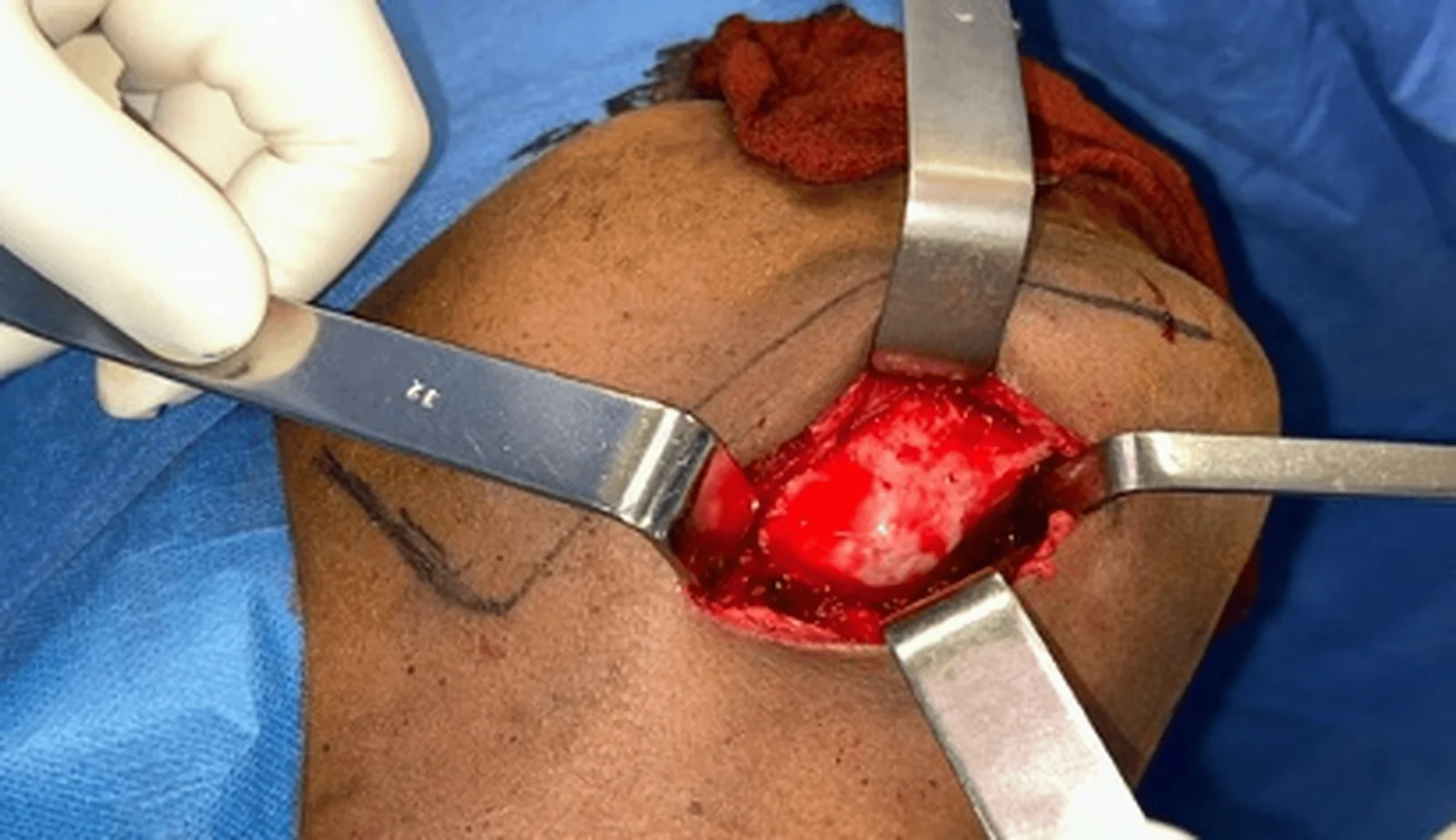
Intraoperative view showing surgical exposure of the right mandible before cortical fenestration. Intraoperative view showing surgical exposure of the right mandibular body after elevation of the mucoperiosteal flap. Marked expansion and thinning of the external mandibular cortex are visible, reflecting the expansive nature of the underlying intraosseous lesion. Cortical fenestration was subsequently performed to gain access to the intraosseous lesion.

**Figure 4:**
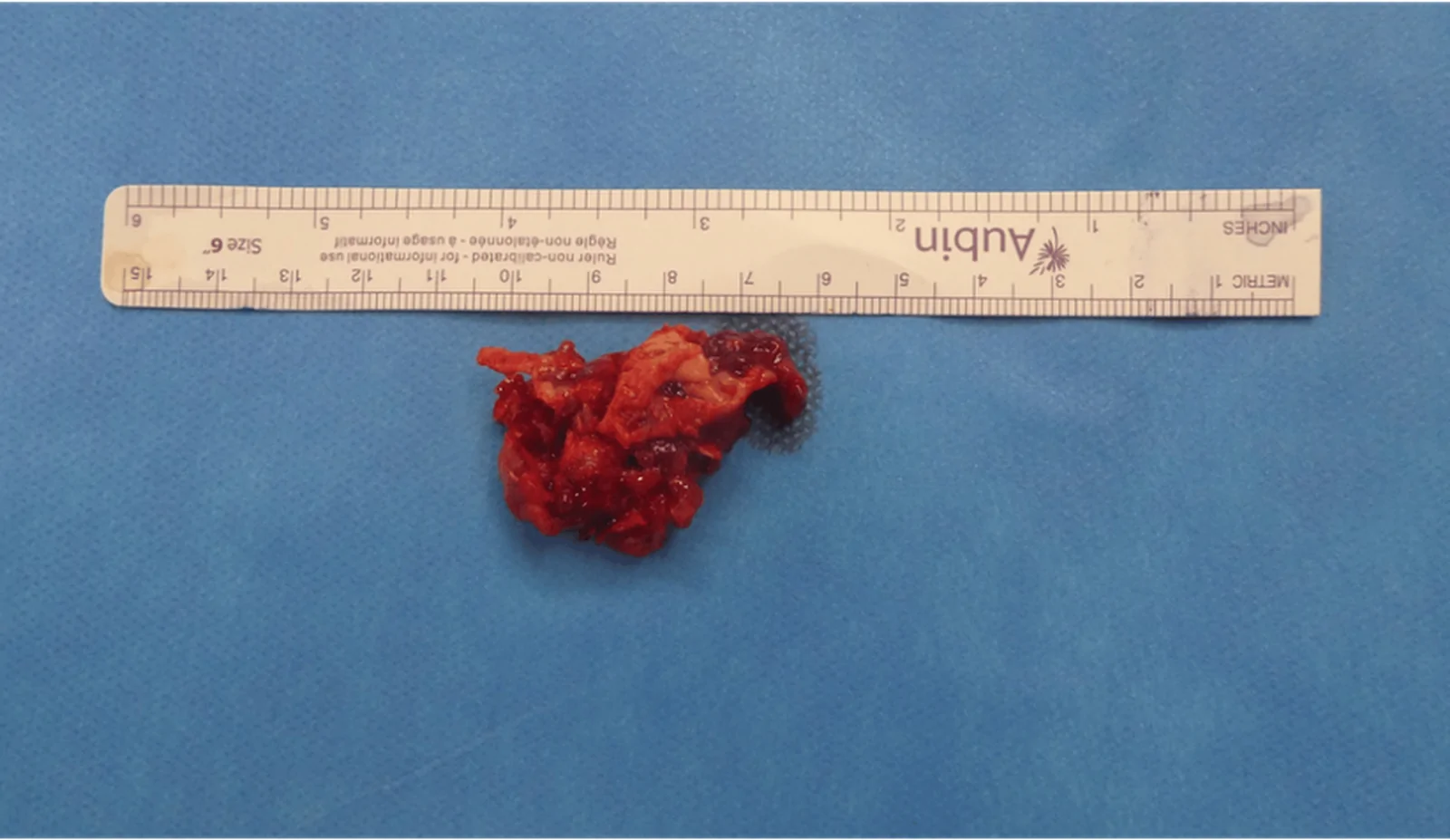
Macroscopic appearance of the right mandibular lesion. Macroscopic appearance of the excised specimen submitted for histopathological examination.

The intramandibular tumor and mandibular bone specimens demonstrated comparable findings. Histological examination revealed a neoplastic proliferation with cartilaginous differentiation organized into nodules. The chondrocytes showed moderate cytological atypia. Numerous binucleated cells were observed, together with enlarged hyperchromatic nuclei and irregular nuclear contours. Part of the lesion was necrotic. No obvious osteoid formation was identified. Importantly, the lesion infiltrated between preexisting bone trabeculae. A small peripheral cystic lesion lined by epithelial cells with strongly eosinophilic cytoplasm was also observed. Its relationship to the main tumor remained uncertain. Immunohistochemical analysis was performed on decalcified material. SATB2 highlighted osteocytes and osteoblasts within infiltrated bone trabeculae. S100 protein staining was negative. KL1 highlighted epithelial cells lining the cystic lesion. The Ki-67 proliferation index was low, within the limitations associated with decalcified material. The initial pathological conclusion was an infiltrative tumor with cartilaginous differentiation suspicious for chondrosarcoma. The pathological material was referred for expert review at the Institut Bergonié, Bordeaux, France, a specialized sarcoma center. Following expert review, the final diagnosis retained was mandibular chondrosarcoma.

Staging investigations

The patient underwent complementary staging investigations. Cervicofacial CT demonstrated postoperative changes related to mandibular surgery. A cluster of right submandibular lymph nodes with preserved fatty hila was observed. No other cervical lymphadenopathy was identified. PET/CT demonstrated mild hypermetabolism of the expansile lytic lesion involving the right mandibular angle and horizontal branch, corresponding to the primary lesion. No distant hypermetabolic lesions were identified. The final staging was T4N0M0.

Definitive surgical treatment

Following multidisciplinary discussion, definitive surgical management was undertaken. The patient underwent right interruptive mandibulectomy with tracheostomy and immediate reconstruction using a vascularized free fibula flap. The mandibulectomy specimen was received fresh and oriented according to its superior, inferior, anterior, and posterior aspects. It measured 7 × 6 × 2.5 cm and included a cutaneous flap measuring 4.5 × 1 cm.

Microscopic examination demonstrated a poorly circumscribed osseous and desmoplastic lesion composed of well-formed bone trabeculae separated by spindle cells arranged in a fascicular pattern. The spindle cells displayed elongated nuclei without significant cytological atypia. The bone trabeculae were arranged in parallel and were focally curved and anastomosing. Despite the relatively bland cytological appearance, the lesion was locally aggressive and infiltrated the mandible, with extension into peripheral striated muscle and fibro-adipose tissue. No tumor emboli were identified. No perineural invasion was observed. Review of the initial biopsy confirmed cartilaginous differentiation. The morphological appearance of the definitive specimen raised the differential diagnosis of low-grade parosteal osteosarcoma.

Immunohistochemical examination demonstrated that the spindle tumor cells were positive for actin and negative for desmin, S100 protein, SOX10, STAT6, CD34, and cytokeratin AE1/AE3. Beta-catenin overexpression was not observed. The Ki-67 proliferation index was approximately 2%. MDM2 and CDK4 testing was not available. Given the discordance between the initial and definitive specimens, expert sarcoma pathology review was crucial. The final diagnosis retained following expert review was mandibular chondrosarcoma. Three right level IIb lymph nodes were examined and demonstrated reactive hyperplasia without evidence of malignancy. The right submandibular gland showed no evidence of malignancy. The anterior and posterior osseous margins were free of tumor. The internal lateral margin measured 0.2 mm, while the external lateral margin measured 2.5 mm.

Postoperative course

Following definitive surgery, the patient was admitted to the intensive care unit for postoperative monitoring. No surgical complications occurred. Serial Doppler monitoring of the vascularized free fibula flap demonstrated satisfactory perfusion without evidence of flap compromise. The patient required transfusion of two units of packed red blood cells because of microcytic anemia identified during the preoperative assessment. Multimodal analgesia provided effective postoperative pain control. The postoperative course was also marked by transient rhabdomyolysis, with a peak creatine phosphokinase concentration of 1,300 IU/L. The laboratory abnormalities progressively improved, and no clinical or biological evidence of compartment syndrome was observed. Ventilatory support was rapidly discontinued. The tracheostomy cuff was deflated before discharge from the intensive care unit. The patient was transferred to the otorhinolaryngology department for further postoperative management and was subsequently discharged home.

## Discussion

Chondrosarcoma is a heterogeneous malignant bone tumor characterized by cartilaginous differentiation. Although chondrosarcoma is among the most common primary malignant bone tumors, involvement of the head and neck remains uncommon [2,6]. Chondrosarcoma affecting the jaw bones is particularly rare. In the largest literature review focused specifically on jaw lesions, 224 cases were identified. Most patients were diagnosed during the second to fifth decades of life; there was a slight predominance in males, swelling was the most frequent presenting symptom, and the maxilla was more commonly affected than the mandible [1]. Earlier series of chondrosarcomas involving the jaw and facial bones similarly described these tumors as rare but locally aggressive [3,4]. The present patient was a 37-year-old woman with mandibular involvement. Although her age was compatible with the age range described in published series, the combination of female sex, mandibular location, and an initial presentation dominated by dental pain and recurrent inflammatory episodes contributed to an atypical clinical presentation.

Clinical presentation and diagnostic delay

Swelling is generally the most frequently reported clinical manifestation of chondrosarcoma of the jaw bones [1]. Other possible manifestations include pain, facial asymmetry, dental displacement, tooth mobility, malocclusion, and neurological symptoms depending on the anatomical extent of the tumor [2,5]. The present case illustrates the potential for diagnostic confusion in the posterior mandible. The patient’s symptoms were initially localized to the region of tooth 46 and were treated as a dental condition. Endodontic treatment was performed, but the symptoms persisted. The subsequent development of recurrent buccal cellulitis further complicated the clinical picture. The patient presented repeatedly to emergency departments with painful inflammatory episodes, including collections requiring drainage and antibiotic treatment.

Recurrent infection may reasonably reinforce an odontogenic diagnosis. However, persistent symptoms despite appropriate dental treatment should prompt reconsideration of the diagnosis. Progressive bone enlargement, cortical alteration, and facial asymmetry should be regarded as warning signs requiring further imaging and histopathological assessment. Repeated loss to follow-up also contributed to the prolonged clinical course. By the time definitive treatment was performed, the lesion had become sufficiently extensive to produce visible extraoral facial asymmetry and required segmental mandibular resection.

Radiological features

The radiological appearance of chondrosarcoma of the jaw may vary according to the tumor matrix, grade, and anatomical extent. CT is useful for evaluating cortical bone involvement and mineralization, whereas MRI provides information regarding soft-tissue extension and the relationship with surrounding structures [2,5]. The lesion in the present case was radiolucent, approximately 40 mm in its greatest dimension, and characterized by irregular borders and cortical expansion. It involved the right posterior mandible, including the horizontal branch and angle. Odontogenic cysts and tumors can present with the same clinical and radiographic findings as chondrosarcomas. The differential diagnosis of an aggressive mandibular lesion includes odontogenic tumors, giant cell lesions, osteosarcoma, primary intraosseous malignancies, metastatic lesions, and other mesenchymal tumors.

The present case emphasizes the importance of correlating radiological findings with the clinical course. Persistent symptoms despite endodontic treatment, progressive mandibular enlargement, and subsequent facial asymmetry were incompatible with an uncomplicated dental infection. PET/CT demonstrated only mild hypermetabolism of the primary lesion, despite the locally aggressive behavior ultimately demonstrated on pathological examination. No distant hypermetabolic lesion was identified.

Histopathological diagnostic challenges

Histopathological diagnosis of chondrosarcoma requires integration of morphological findings with radiological and clinical information. Major diagnostic considerations include the degree of cytological atypia, binucleation, cellularity, matrix production, and, importantly, the relationship between the tumor and surrounding bone [2,6]. The initial specimen in the present case demonstrated nodular cartilaginous differentiation associated with moderate atypia, numerous binucleated cells, enlarged hyperchromatic nuclei, irregular nuclear contours, and partial necrosis. The absence of obvious osteoid formation was also documented. Importantly, the lesion infiltrated between preexisting bone trabeculae. Infiltrative growth is an important feature supporting malignant behavior in cartilaginous tumors.

Interpretation was complicated by the decalcified nature of the specimen. Immunohistochemical findings must therefore be interpreted cautiously. The absence of S100 protein staining and the low Ki-67 proliferation index did not exclude malignancy in this context. The definitive mandibulectomy specimen demonstrated a different predominant morphology, characterized by an osseous and desmoplastic lesion composed of well-formed bone trabeculae and spindle cells. Although the cytological appearance was relatively bland and the Ki-67 proliferation index was low, the lesion demonstrated aggressive local infiltration of bone, muscle, and fibro-adipose tissue. This morphology raised the differential diagnosis of low-grade parosteal osteosarcoma. Surface osteosarcomas and cartilaginous tumors may demonstrate overlapping morphological characteristics, and distinguishing between these entities may require adequate sampling and expert pathological interpretation.

The discordance between the initial and definitive pathological findings is therefore one of the most relevant aspects of this case. It demonstrates the importance of expert review in rare bone tumors, particularly when different specimens demonstrate different morphological patterns. Following expert sarcoma review at the Institut Bergonié, the final diagnosis retained was mandibular chondrosarcoma.

Differential diagnosis

The differential diagnosis evolved throughout the patient’s clinical course. Initially, an odontogenic or inflammatory lesion was suspected because of localized dental pain, treatment of tooth 46, and recurrent episodes of buccal cellulitis. However, progressive mandibular enlargement and destructive imaging findings were not adequately explained by an uncomplicated odontogenic infection. During the initial surgical procedure, the solid consistency of the lesion argued against a conventional dentigerous or inflammatory cyst. Histologically, benign cartilaginous lesions were considered in the differential diagnosis of the initial specimen. However, moderate atypia, binucleation, nuclear abnormalities, necrosis, and infiltration between preexisting bone trabeculae supported malignant behavior.

The definitive specimen subsequently raised the possibility of low-grade parosteal osteosarcoma because of its desmoplastic spindle-cell morphology and associated bone formation. This diagnostic complexity highlights the necessity of integrating clinical, radiological, and pathological findings rather than relying on a single specimen or isolated morphological feature.

Surgical treatment

Complete surgical resection remains the mainstay of treatment for chondrosarcoma whenever anatomically feasible [1,2,6]. In the present case, the locally advanced mandibular lesion required interruptive mandibulectomy. The definitive specimen confirmed aggressive local behavior, with infiltration of the mandible and extension into striated muscle and fibro-adipose tissue. The anterior and posterior osseous margins were free of tumor. However, the soft-tissue margins were close, particularly the internal lateral margin, which measured 0.2 mm. The surgical management of chondrosarcoma of the head and neck is complicated by anatomical constraints. Achieving oncologically appropriate margins must be balanced against preservation of critical functional and aesthetic structures. Published studies emphasize the importance of local control and complete surgical treatment in the management of chondrosarcoma [1,2,6].

Mandibular reconstruction with a free fibula flap

Segmental mandibular resection produces loss of mandibular continuity and may have major consequences for facial symmetry, mastication, speech, swallowing, and dental rehabilitation. The vascularized free fibula flap is widely used for reconstruction of extensive mandibular defects because it provides a substantial length of vascularized bone that can be osteotomized and adapted to the mandibular contour [7,8]. In the present case, immediate reconstruction using a vascularized free fibula flap was performed following interruptive mandibulectomy. Serial Doppler monitoring demonstrated satisfactory flap perfusion, and no flap-related surgical complication was reported. The use of a vascularized bone flap allowed immediate restoration of mandibular continuity following oncological resection.

Prognosis and follow-up

Chondrosarcoma of the jaw and facial bones is primarily characterized by the risk of local progression and recurrence [1,3,4]. In one clinicopathological series, local recurrence was reported in approximately one-third of patients with adequate follow-up, illustrating the importance of long-term surveillance. Prognosis is influenced by factors including histological characteristics, local recurrence, metastatic disease, and the adequacy of treatment [1,6,9]. In the present case, staging investigations revealed no evidence of distant metastatic disease. Cervicofacial imaging did not demonstrate suspicious cervical lymphadenopathy, PET/CT showed no distant hypermetabolic lesions, and three right level IIb lymph nodes were histologically negative for malignancy. The tumor was classified as T4N0M0. Long-term multidisciplinary follow-up is essential because of the risk of local recurrence, particularly in the context of a locally advanced lesion and close soft-tissue margins.

## Conclusions

Mandibular chondrosarcoma is a rare malignant bone tumor that may initially mimic odontogenic or inflammatory conditions. In this case, recurrent episodes of mandibular pain, initial endodontic treatment, and recurrent buccal cellulitis contributed to a misleading clinical presentation. Progressive mandibular enlargement, facial asymmetry, and destructive radiological changes should prompt investigation for an underlying neoplasm. While clinical and radiological findings raised concern for an aggressive lesion, the unexpected intraoperative discovery of a solid tumor was a major turning point, confirming that the lesion was not consistent with a simple odontogenic infection.

The discordant histopathological findings further contributed to the diagnostic complexity, and expert sarcoma pathology review was decisive in establishing the definitive diagnosis of chondrosarcoma. Definitive management required an interruptive mandibulectomy with immediate reconstruction using a vascularized free fibula flap. This case highlights the complementary roles of clinical assessment, imaging, surgical findings, and specialized pathology in the diagnostic process and management of rare mandibular tumors.
